# Physicochemical, Volatile Compound Profile, Antioxidant, and Cytotoxic Activities of Northeastern Thai Ethnic Ready-to-Serve Food Pastes Jaew Hon and Gang Om: A Comparative Study of Laboratory and Industrial Production Processes

**DOI:** 10.3390/foods14050876

**Published:** 2025-03-04

**Authors:** Vijitra Luang-In, Worachot Saengha, Thipphiya Karirat, Piyathida Promjamorn, Nidthaya Seephua, Apichaya Bunyatratchata, Sudathip Inchuen, Kriangsak Banlue, Sarinthorn Suwannarong, Sirithon Siriamornpun

**Affiliations:** 1 Natural Antioxidant Innovation Research Unit, Department of Biotechnology, Faculty of Technology, Mahasarakham University, Khamriang, Kantarawichai, Maha Sarakham 44150, Thailand; vijitra.l@msu.ac.th (V.L.-I.); worachot207@gmail.com (W.S.); thipphiya.k@gmail.com (T.K.); piyathida.pprom@gmail.com (P.P.); 2 Research Unit of Thai Food Innovation, Department of Food Technology and Nutrition, Mahasarakham University, Khamriang, Kantarawichai, Maha Sarakham 44150, Thailand; nittayaseepua1@gmail.com (N.S.); apichaya.b@msu.ac.th (A.B.); sudathip.i@msu.ac.th (S.I.); kriangsak.b@msu.ac.th (K.B.); sarinthorn.s@msu.ac.th (S.S.)

**Keywords:** Thai culinary paste, herbal ethnic food, phytochemicals, MCF-7, HT-29, HSC-7

## Abstract

Northeastern Thai ethnic foods are celebrated for their health benefits yet remain largely underexplored. This study assessed the antioxidant and cytotoxic properties of two ready-to-eat pastes—Jaew Hon (JH) and Gang Om (GO)—produced using laboratory (LAB) and industrial original equipment manufacturer (OEM) methods. Evaluations were conducted using 2,2-Diphenyl-1-picrylhydrazyl (DPPH), ferric reducing antioxidant power (FRAP), total phenolic content (TPC), and total flavonoid content (TFC) assays alongside the 3-(4,5-Dimethylthiazol-2-yl)-2,5-diphenyltetrazolium bromide (MTT) assay for cytotoxicity. Physicochemical analyses revealed that JH OEM had the highest total dissolved solids (11.57°Brix) and water activity (0.91), while GO OEM exhibited the highest pH (5.28) and lightness (L* 31.43). Antioxidant results showed JH LAB outperformed in DPPH scavenging (96.25 mg AAE/100 g) and TPC (433.5 mg GAE/100 g), whereas GO OEM achieved the highest TFC (345.57 mg QE/100 g). Volatile compound profiling by Gas Chromatography–Mass Spectrometry (GC-MS) indicated distinct aroma profiles between LAB and OEM samples. Moreover, MTT assays revealed stronger cytotoxic effects for OEM products; specifically, GO OEM achieved 71.88% maximum inhibition and an IC50 of 276.10 µg/mL against HT-29 cells. Colony formation assays confirmed GO OEM’s significant antiproliferative activity, and gene expression analysis demonstrated upregulation of pro-apoptotic markers (*Bax*, *Caspase-3*) alongside downregulation of *NF-κB p65*, *Cyclin D1*, and *MMP-9*. Overall, these findings suggest that industrially produced GO and JH pastes hold promise as functional foods, integrating traditional culinary practices with modern production techniques. These findings lay the foundation for future research focused on uncovering bioactive mechanisms, optimizing processing methods, and confirming health benefits through in vivo studies.

## 1. Introduction

Isan cuisine, emblematic of northeastern Thailand’s local food culture, is renowned for its distinctive flavors and aromas derived from natural, locally sourced ingredients. This regional culinary tradition is marked by its inherent simplicity, characterized by the minimal use of condiments and straightforward cooking methods that mirror the agricultural lifestyle and the brisk pace of life in the region. The gastronomic repertoire of Isan includes a diverse array of dishes such as richly spiced curries (Gang Om), aromatic soups (Tom), flavorful meat salads (Larb or Koi), tangy dips (Jaew), and an assortment of wrapped, steamed, or roasted preparations (Mok), in addition to various salads like papaya salad (Som Tam) [1,2]. Sugar use in Isan cuisine is typically limited, with sweetness often achieved through natural ingredients, further emphasizing its reliance on fresh and local produce [2]. Isan cuisine is increasingly recognized for its potential health benefits. The integration of natural ingredients—including a variety of medicinal herbs and spices—imbues Thai culinary traditions with natural bioactives known for their antibacterial, anti-inflammatory, antidiabetic, anticancer, hepatoprotective, and cardioprotective properties [3]. These health-promoting attributes have spurred a growing global interest in functional foods derived from ethnic culinary traditions.

The bioavailability of minerals and phytochemicals in food is significantly enhanced by modern food processing techniques, particularly retort processing. This high-temperature sterilization method (110–121 °C), conducted in sealed containers, not only ensures food safety and extends shelf life but also facilitates the breakdown of complex food matrices, improving nutrient digestibility and increasing antioxidant availability [4]. For instance, in black beans, retort processing enhances nutrient absorption, while in muskmelon puree, it increases the occurrence of volatile molecules, such as esters, which contribute to both flavor and bioactive properties [5,6].

In industrial food production, optimizing these processes is crucial to maintaining a balance between taste, nutrition, and bioactivity. Large-scale production often relies on original equipment manufacturer (OEM) technologies to ensure consistency and scalability in ready-to-serve products, whereas laboratory-scale (LAB) production allows for precise control over processing parameters but remains limited in volume. Despite the widespread application of thermal processing in the food industry, comparative studies on its effects, particularly on traditional Thai food pastes, remain scarce. Understanding these impacts could further unlock the potential of cooking techniques to improve the nutritional value and functional properties of food.

Previous research has explored the nutritional and bioactive potentials of individual ingredients in Thai cuisine [7,8,9]; however, there remains a noticeable gap in studies directly comparing the effects of laboratory-scale (LAB) versus original equipment manufacturer (OEM) production processes on traditional ethnic food pastes. This comparative analysis is particularly relevant for products like Jaew Hon (JH) and Gang Om (GO), which are gaining popularity both locally and internationally [10]. While these pastes have traditionally been prepared using time-honored methods, the adaptation of modern processing techniques raises important questions about potential changes in their physicochemical properties, volatile compound profiles, antioxidant capacities, and cytotoxic activities.

For instance, retort processing has been successfully applied to southern-style Pad Thai sauce, ensuring shelf stability while preserving its distinct qualities. The incorporation of potato starch and xanthan gum provided structural stability, while mixed antioxidants enhanced oxidative resistance, making it a high-quality and convenient product for broader markets [11]. In addition, thermal processing (121 °C, 15 psi, 20 min) enhanced the functionality of ready-to-eat ricebean curry by improving protein digestibility, calcium bioavailability, and antioxidant activity [12]. The optimized formulation retained bioactive compounds like polyphenols, flavonoids, and lycopene, contributing to its nutritional and sensory quality. Moreover, thermal processing influenced the bioactive compounds and physicochemical stability of ready-to-cook Thai sauces, with sterilization at 121 °C (43 min) for stir-fry curry sauce (SCS) and 102 °C (31 min) for spicy-sour sauce (SSS). SSS had higher phenolics, carotenoids, and antioxidant capacity, while storage at 55 °C resulted in greater reductions in lightness, water activity, and pH over 90 days. This highlighted the role of thermal processing in improving the stability and bioactivity of traditional food products [13].

Thus, this study aims to bridge this research gap by systematically evaluating the effects of LAB and OEM production processes on two traditional northeastern Thai food pastes, JH and GO. By employing a comprehensive analytical framework that encompasses physicochemical assessments, volatile compound profiling, and evaluations of antioxidant and cytotoxic properties, the investigation aims to elucidate the influence of production scale on the quality and bioactivity of these products. Analytical assays, including 2,2-Diphenyl-1-picrylhydrazyl (DPPH), ferric reducing antioxidant Power (FRAP), total phenolic content (TPC), total flavonoid content (TFC), and the 3-(4,5-Dimethylthiazol-2-yl)-2,5-diphenyltetrazolium bromide (MTT) assay, were employed to generate high-resolution data that capture both the nutritional and functional profiles of the pastes.

The rationale for this study is multifaceted. First, there is a pressing need to elucidate how industrial processing affects the functional attributes of traditional ethnic foods in an era where convenience is paramount, yet consumers remain increasingly health-conscious. While industrial methods such as OEM production offer the advantages of scalability and product consistency, they may inadvertently alter the bioactive composition and sensory qualities that define traditional pastes. A direct comparison with LAB-produced samples provides an opportunity to pinpoint critical processing parameters that can be optimized to preserve—or even enhance—the desirable attributes of these foods. Second, by focusing on JH and GO, the study contributes to the burgeoning field of functional foods, which has gained substantial momentum as a strategy to combat modern health challenges. Understanding the interplay between traditional culinary practices and contemporary processing technologies is essential for developing next-generation food products that are both culturally authentic and scientifically validated. The successful integration of modern production methods with traditional culinary wisdom not only enhances the market potential of these pastes but also contributes to preserving the culinary heritage of northeastern Thailand.

The aims of this work are threefold: (1) to compare the physicochemical, volatile compound, antioxidant, and cytotoxic properties of JH and GO produced by LAB and OEM methods; (2) to elucidate the influence of these production processes on the functional characteristics of the pastes; and (3) to establish a scientific basis for optimizing production techniques that can yield ready-to-serve products with enhanced bioactivity and cultural authenticity. This research endeavors to bridge the gap between traditional culinary practices and modern food technology, thereby paving the way for further studies aimed at uncovering the mechanistic pathways underlying the bioactivity of ethnic food products. Ultimately, such efforts are expected to lead to the creation of safer, more effective functional foods that cater to contemporary health demands while honoring traditional heritage.

## 2. Materials and Methods

### 2.1. Laboratory Production Process

**Figure 1:**
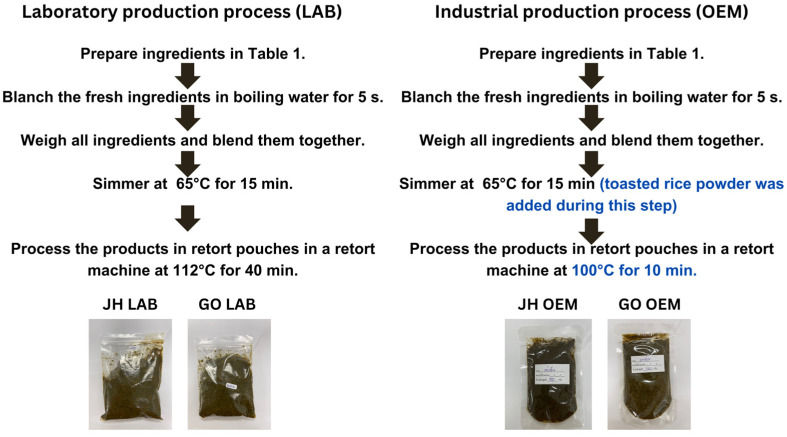
Laboratory and industrial production processes and appearance of four pastes. Jaew Hon lab scale (JH LAB), Jaew Hon industrial scale (JH OEM), Gang Om lab scale (GO LAB), and Gang Om industrial scale (GO OEM). Blue fonts indicate the differences in the production processes.

All of the raw ingredients (Table 1) were obtained from a local market in Muang Mahasarakham District, Mahasarakham Province, Thailand, and were thoroughly washed to ensure cleanliness prior to blanching in boiling water for 5 s. Subsequently, the blanched ingredients were rapidly cooled in ice-cold water to halt the cooking process and preserve their freshness. The ingredients were blended to a paste and simmered at 65 °C for 15 min. Once cooked, the products were immediately packaged into 75 mL pouches. Subsequently, these were subjected to thermal processing in a stainless steel retort processing device (SR Advance, Nonthaburi, Thailand) using a hot water–air mixture for sterilization at 112 °C for 40 min with pressure maintained at 15 psi (Figure 1).

### 2.2. Industrial Production Process

The same was carried out as above. However, the following steps were conducted by the original equipment manufacturer (OEM). Subsequently, the blanched ingredients were rapidly cooled in ice-cold water to halt the cooking process and preserve their freshness. All ingredients (Table 1), except for the toasted rice powder, were blended into a fine mixture paste and simmered at 65 °C for 15 min, during which the toasted rice powder was added. Once cooked, the products were immediately packaged while still hot into 100 mL pouches. They were subsequently subjected to thermal processing in a stainless steel retort processing device (All American, Redmond, OR, USA) using a hot water–air mixture for sterilization at 100 °C for 10 min with pressure maintained at 15 psi (Figure 1).

### 2.3. Physicochemical Analysis

The pH values of the pastes were measured with a pH meter (CLEAN pH30 Tester, Clean Instrument, Shanghai, China). Water activity (aw) was assessed utilizing an Aqua Lab water activity meter (METER, Pullman, WA, USA). The color characteristics were assessed using a Chroma Meter CR-400 (Konica Minolta, Tokyo, Japan). Approximately 6 g of each sample was placed into a 35 mm diameter Petri plate for analysis. The L* value represents the lightness of the sample, ranging from 0 to 100 (black to white). The a* value reflects the level of redness, where positive a* indicates red and negative a* signifies green. Meanwhile, the b* value denotes yellowness, with positive b* indicating yellow and negative b* representing blue. Total dissolved solids (TDS) were quantified in degrees Brix utilizing a handheld refractometer (Atago, Tokyo, Japan).

### 2.4. Antioxidant Activity and Bioactive Content

#### 2.4.1. Sample Preparation and Extraction

All four paste samples (Figure 1), JH LAB, JH OEM, GO LAB, and GO OEM, in triplicate, were oven-dried at 42 °C for 48 h. All dried materials were pulverized into powders, then extracted with 80% ethanol (1:10 ratio), agitated in darkness at 25 °C for 24 h, and then separated using vacuum suction with a Buchner funnel. This was followed by centrifugation at 4 °C for 20 min at 7100× *g*. The ethanolic extract was then concentrated using an evaporator and subsequently stored at −20 °C until needed.

#### 2.4.2. 2,2-Diphenyl-1-picryl hydrazyl (DPPH) Radical Scavenging Activity

This was determined using a modified version of the previously described methodology [14,15]. Initially, 100 µL of the sample extract (20 mg/mL in 80% ethanol) was mixed with 100 µL of 0.2 mM DPPH in 95% ethanol. The mixture was then incubated in darkness at 30 °C for 30 min. Finally, the absorbance was measured at 517 nm, with L-ascorbic acid equivalent (AAE) serving as the standard reference.

#### 2.4.3. Ferric Reducing Antioxidant Power (FRAP)

The FRAP assay was performed following modified protocols [14,15]. A freshly prepared FRAP solution, consisting of 300 mM acetate buffer (pH 3.6), 10 mM 2,4,6-tripyridyl-s-triazine (TPTZ) in 40 mM HCl, and 20 mM FeCl_3_•6H_2_O in a 10:1:1 ratio, was incubated at 37 °C for 30 min. Then, 15 µL of the paste extract (20 mg/mL) was mixed with 285 µL of the FRAP solution and further incubated at 37 °C for 30 min. The absorbance was measured at 593 nm using FeSO_4_ as the standard reference.

#### 2.4.4. Total Phenolic Content (TPC)

TPC was determined using a modified version of the previously described method [14,15]. Briefly, 20 µL of the paste extract (20 mg/mL) was mixed with 100 µL of 10% Folin-Ciocalteu reagent (*v*/*v*). After incubating in darkness at 37 °C for 6 min, 7.5% anhydrous Na_2_CO_3_ (*w*/*v*) was added, and the mixture was further incubated for 30 min. The absorbance was measured at 765 nm, with TPC expressed as gallic acid equivalent (GAE).

#### 2.4.5. Total Flavonoid Content (TFC)

TFC was assessed following a modified version of the previously described method [14,15], with adjustments in volume and the use of a 96-well plate instead of a test tube. Briefly, 20 µL of the paste extract (20 mg/mL) was mixed with 60 µL of distilled water in a 96-well plate, followed by the addition of 10 µL of a 5% NaNO_2_ solution. After 6 min, 10 µL of a 10% AlCl_3_·6H_2_O solution was added and allowed to stand for 5 min. Then, 100 µL of 1 M NaOH was added, and the mixture was incubated for 30 min. The absorbance was measured at 420 nm, with quercetin (QE) used as the standard reference.

### 2.5. GC-MS Analysis of Volatile Compounds

The analysis used an Agilent 7890A gas chromatograph with an HP-5MS capillary column (30 m × 0.25 mm, fused silica, 0.25 µm) and an Agilent 7000B mass spectrometer (Agilent Technologies, Santa Clara, CA, USA). Data were processed using Agilent Mass Hunter Qualitative Analysis Workstation version 10 and the NIST MS Search 2.0 library. Solid-phase microextraction (SPME) was performed on a 1-gram paste sample in a headspace vial with a 50/30 µm DVB/CAR/PDMS fiber. Autosampler conditions included 5 min pre-incubation, 15 min incubation and extraction at 100 °C, and 5 min desorption. Scan mode mass spectrometry (mass range: 35–500) was employed with electron ionization (EI) at 70 eV and ion source set at 230 °C. Split injection mode (5:1) at 250 °C with helium (flow rate of 1.0 mL/min) and 250 °C transfer line temperature was used. The column oven temperature was programmed as follows: initially set at 60 °C (no hold), then increased to 200 °C at a rate of 3 °C/min with a 3-min hold, followed by a further increase to 280 °C at 10 °C/min with another 3-min hold, for a total run time of 60.667 min. The total ion current chromatogram’s respective peak areas determined semi-quantification. Only compounds with 80% probability were selected for results to balance identification accuracy and data comprehensiveness. This cutoff was chosen to minimize false positives and reduce the risk of compound misclassification due to spectral similarities. While a lower threshold could increase the number of identified volatiles, it may compromise reliability. Online SRPlot [16] was used to develop a heatmap and dendrogram of volatile chemicals in the four pastes.

### 2.6. Cancer Cell Cultivation and Cytotoxicity Determination Using MTT Assay

The HSC-7 oral cancer cells were obtained from the Health Science Research Resources Bank (HSRRB) in Japan, while the HT-29 colon cancer cells and MCF-7 breast cancer cells were sourced from the American Type Culture Collection (ATCC) in Manassas, VA, USA. As previously described [17], they were cultivated in Dulbecco’s Modified Eagle’s Medium (DMEM) supplemented with 10% fetal bovine serum (Gibco, Thermo Fisher Scientific, Inc., Waltham, MA, USA). The cytotoxicity of each paste was assessed against three cancer cell lines. Five thousand cells/well were seeded in 96-well plates overnight. Subsequently, cells were subjected to a range of crude extract concentrations (0–800 µg/mL) for a 24-h period. The extract concentrations in the MTT assay were selected based on preliminary screening and previous work [17] to capture dose-dependent effects and determine IC_50_ values while maintaining cell viability. Five thousand cells/well were optimized to ensure proper adherence, growth, and consistency, preventing excessive confluency and minimizing variability in metabolic activity measurements. The medium was replaced with MTT reagent and incubated for 4 h. The resulting formazan crystals were dissolved in 200 µL of DMSO, and the absorbance at 590 nm was measured in triplicate. The IC_50_ values and cytotoxicity (%) were evaluated at 24 h. The chemicals were all supplied by Sigma-Aldrich, a company located in St. Louis, MO, USA.

### 2.7. Antiprolifeative Activity Using Clonogenic Formation Assay

The anti-colony formation efficacy of the paste extracts was evaluated. HT-29 cells (500 cells/well) were seeded into 6-well plates and incubated overnight. The cells were then maintained at 37 °C with 5% CO_2_ and treated with paste extract at concentrations of 0, 400, and 800 µg/mL in fresh medium. After treatment, the cells were washed twice with phosphate-buffered saline (PBS) and cultured in fresh DMEM for an additional 14 days, with the medium being replaced every 3 days. Following the incubation period, the cells were rinsed with PBS and fixed with methanol for 1 h. The colonies were stained with 0.5% Coomassie Brilliant Blue G-250 dissolved in methanol for 30 min, after which the excess stain was washed off with water. The colony formation was quantified using ImageJ software version 1.54i, ensuring accurate counting and analysis. A colony was defined as a cluster of at least 50 cells, following established criteria in clonogenic assays. The percentage of colony formation was calculated as follows:

Colony formation (%) = (Number of colonies in treated group/Number of colonies in control group) × 100.

IC_50_ values were calculated using GraphPad Prism version 9.0 with non-linear regression analysis.

### 2.8. Apoptosis-Related Gene Expression Using Real-Time Polymerase Chain Reaction

The expression of apoptosis-related genes in HT-29 cells was assessed using SYBR Green-based real-time PCR following treatment with GangOm (OEM) and JaewHon (OEM) at concentrations of 0, 25, 50, and 100 µg/mL for 24 h. RNA was extracted using the easy-spin™ reagent (Intron Biotechnology, Seongnam-si, Republic of Korea), and reverse-transcribed into cDNA using iScript™ Supermix (BIO-RAD, Hercules, CA, USA). Real-time PCR was performed on a CFX Duet system using PowerTrack SYBR Master Mix (Thermo Fisher Scientific, Waltham, MA, USA) and specific primers (Table 2). The 2^−^∆∆CT method was used for statistical analysis of CT values, with *GAPDH* serving as the housekeeping gene for normalization. Triplicate measurements were conducted. The real-time PCR conditions were as follows: denaturation at 94 °C for 10 min, annealing at 60 °C for 10 s, and extension at 72 °C for 10 s, for a total of 45 cycles.

### 2.9. Protein Expression by Western Blot Assay

HT-29 cells (5 × 10^5^ cells/well) were treated with plant extract for 24 h, lysed using RIPA buffer (Intron Biotechnology, Seongnam-si, Republic of Korea) containing protease and phosphatase inhibitors (Sigma-Aldrich, St. Louis, MO, USA), and centrifuged at 12,000× *g* for 15 min at 4 °C. Protein concentration was measured using a BCA assay reagent (Thermo Fisher Scientific, Waltham, MA, USA). Equal amounts of protein (20 µg) were separated via 12% SDS-PAGE, transferred onto PVDF membranes (Millipore, Burlington, MA, USA), blocked with 5% milk in TBST, and incubated overnight at 4 °C with primary antibodies (anti-NF-κB p65 and anti-GAPDH, 1:5000) (Abcam, Cambridge, UK). Before visualization with an ECL substrate (Millipore, Burlington, MA, USA), membranes were incubated with biotinylated secondary antibodies and HRP-conjugated streptavidin (Thermo Fisher Scientific, Waltham, MA, USA). Densitometric analysis was performed using ImageLab software version 6.1 (BIO-RAD, Hercules, CA, USA), and protein bands were captured using a ChemiDoc System (BIO-RAD, Hercules, CA, USA). NF-κB p65 levels were normalized to GAPDH.

### 2.10. Statistical Analysis

One-way ANOVA and Duncan’s multiple range test (SPSS version 27, IBM, Armonk, NY, USA) were used to determine the means and standard deviations (SD) of triplicate experimental data at a significance level of *p* < 0.05. Pairwise differences between groups were evaluated using post hoc multiple comparisons with Tukey’s test. The statistical significance criteria were set as follows: *p* < 0.05 (*), *p* < 0.01 (**), *p* < 0.001 (***), and *p* < 0.0001 (****).

## 3. Results and Discussion

### 3.1. Physicochemical Attributes of the Four Pastes in Laboratory and Industrial Production

The physicochemical attributes of the four pastes produced in both laboratory and industrial settings reveal significant differences that can influence their applications in food production (Table 3).

The pH values ranged from 4.92 to 5.28, reflecting a slightly acidic environment across all samples. The lower pH observed in the LAB samples can be attributed to the more intense thermal conditions (higher temperature and longer duration), which likely facilitated the formation of Maillard Reaction Products (MRPs) through interactions between proteins, reducing sugars, and herbal extracts [11,18], resulting in reduced pH levels [11]. Additionally, the greater use of herbal ingredients in JH may have contributed to the lower pH, as herbal extracts are mildly acidic due to the presence of natural acids [11]. Similarly, the TDS measurements, ranging from 6.67 to 11.57°Brix, indicate that JH has higher TDS values, likely attributed to the greater quantity of herbal ingredients used. However, thermal conditions in OEM production preserved soluble solids such as sugars and salts, resulting in higher TDS values than the LAB setting. GO OEM had the highest water activity (aw) of a_w_ (0.94), and JH LAB had the lowest aw of a_w_ (0.90).

Color attributes, represented by L*, a*, and b* values, also varied significantly among the samples. The GO OEM paste had the highest L* value (31.43), indicating a lighter color, while the JH OEM paste had the lowest L* value (29.57), suggesting a darker appearance. The milder OEM thermal processing may reduce Maillard reactions and caramelization, preserving the lighter color of the paste [18]. In contrast, extended heat exposure in LAB samples intensified these browning reactions, leading to darker pastes. Maillard browning can be mitigated by reducing moisture, pH, or temperature, particularly in liquid-based products [18]. Heat also impacts natural pigments in herbs, such as chlorophylls, anthocyanins, and carotenoids, with anthocyanins undergoing browning. While traditional retorting often alters these pigments due to extended heating, high-temperature short-time processes help minimize such changes [18]. Additionally, the red–green (a*) and yellow–blue (b*) color parameters varied, with JH OEM displaying the highest a* value (1.82), likely due to the better preservation of pigments such as carotenoids under milder OEM conditions [11]. The importance of processing conditions in determining the quality and visual appeal of the pastes is underscored by these findings.

### 3.2. Antioxidant Activity and Bioactive Content Analysis

The assessment of key herbal ingredients revealed that chili, kaffir lime leaves, and shallot exhibited the highest DPPH scavenging activity (Table 4).

These results are consistent with the existing literature, which highlights the potent antioxidant properties of these botanicals. These properties are attributed to their dense phytochemical profiles, which include flavonoids and phenolic compounds that are recognized for their free radical scavenging capabilities [19,20,21]. Generally, the OEM process enhanced antioxidant properties and bioactive contents. Both the highest TPC and TFC contents were found in GO OEM, followed by JH OEM (Table 5). The TPC value is strongly correlated to DPPH scavenging activity. Phenolic compounds contribute to reducing power, but other non-phenolic compounds (e.g., vitamin C, some carotenoids) can also influence the FRAP values, diluting the strength of the correlation of FRAP with TPC.

Previously, the retort process at 121 °C enhanced the antioxidative activity of herbal hydrolyzed collagen soups by promoting the formation of MRPs and interactions with phenolic compounds, which act as effective antioxidants [11]. Flavonoids and phenolic compounds in fruits and vegetables are influenced by food processing [22,23]. Heat treatment, such as converting tomatoes into paste, increases total phenolics and flavonoids due to enhanced extractability from cell structure rupture during thermal processing. Longer heat exposure further boosts these levels [24]. This may explain why, sometimes, the LAB setting increased TPC and DPPH antioxidant activity. The outcomes can be influenced by sample matrix and interfering components, such as other reducing agents in the Folin-Ciocalteu assay or non-flavonoid polyphenols in the aluminum chloride method [25,26]. Overall, variations in the types and proportions of herbal ingredients between paste formulas (JH vs. GO), along with differences in production methods (LAB vs. OEM), led to distinct antioxidant activities and bioactive contents. The health benefits of the pastes are, in turn, greatly affected by these differences.

### 3.3. Volatile Compound Profile

The three most abundant volatile compounds in each sample reveal distinct chemical profiles influenced by production methods (Table 6). In JH OEM, myristicin (24.50%), beta-caryophyllene (17.31%), and sorbic acid (7.52%) dominate, while JH LAB features selina-6-en-4-ol (7.99%), beta-caryophyllene (8.58%), and estragole (6.67%). Similarly, in GO OEM, the top compounds are sorbic acid (18.81%), myristicin (18.09%), and beta-caryophyllene (5.89%), whereas GO LAB highlights selina-6-en-4-ol (17.02%), beta-caryophyllene (6.31%), and myristicin (4.27%). These results show a trend where myristicin, sorbic acid (as preservative), and estragole are predominant in industrial samples, while selina-6-en-4-ol is more abundant in laboratory-prepared pastes. Notably, beta-caryophyllene consistently ranks among the top compounds across all samples, highlighting its stability and importance in the volatile profiles. Interestingly, myristicin, beta-caryophyllene, and selina-6-en-4-ol have documented anticancer activities [27,28,29,30,31,32].

Linked to an inhibitory effect on HepG2 and A549 cell growth, selina-6-en-4-ol induces apoptosis and stops the cell cycle at the G1 phase. The alteration of mitochondrial membrane potential and the increase of the p21 protein are likely related to this activity [18]. Through mechanisms that include nuclear condensation and fragmentation and the alteration of mitochondrial membrane potential, β-caryophyllene initiates apoptosis. Additionally, it exhibits strong inhibitory effects on colon cancer cells by suppressing clonogenic growth, migration, invasion, and spheroid formation [33].

Consistent compounds, such as acetic acid and beta-linalool, suggest some volatiles are unaffected by production methods. Differences likely stem from process variations, ingredient handling, and industrial efficiency, influencing the retention or loss of certain volatiles. These variations can significantly affect sensory attributes, such as the citrus-like aromas enhanced by increased citronellol and citronellal or the diminished nutmeg-like notes due to reduced myristicin. These findings underscore the importance of production optimization to achieve desirable flavor profiles and consistency across scales.

**Figure 2:**
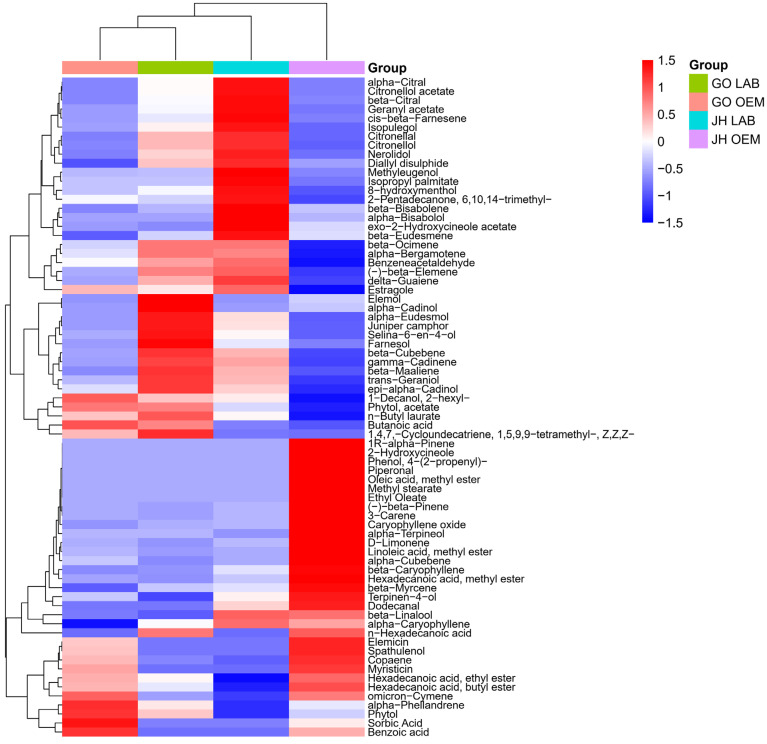
Heatmap with dendrogram showing clustering of four pastes and volatile compounds. GO = Gang Om; JH = Jaew Hon; LAB = laboratory production; OEM = industrial production. Relative abundance of each compound is scaled from low (blue) to high (red).

The heatmap reveals distinct differences in the relative abundances of volatile compounds between LAB and OEM production methods for both JH and GO pastes, with hierarchical clustering grouping samples based on their volatile profiles (Figure 2). Selina-6-en-4-ol is notably abundant in LAB samples, particularly in GO LAB, suggesting enhanced preservation or synthesis of this sesquiterpenoid in laboratory settings, while myristicin is significantly more abundant in OEM samples, indicating potential degradation or loss during laboratory processing. Compounds like beta-caryophyllene and estragole appear across all samples, reflecting their stability and ubiquitous presence. LAB samples (GO LAB and JH LAB) form a distinct cluster with higher levels of terpenoids such as citronellol, selina-6-en-4-ol, and gamma-cadinene, whereas OEM samples (GO OEM and JH OEM) cluster separately, dominated by higher levels of sorbic acid and myristicin. These findings highlight that industrial processing enhances the retention of stable compounds like myristicin and sorbic acid, while laboratory methods favor the retention of bioactive terpenoids like selina-6-en-4-ol and citronellol. This observation is consistent with previous research that has demonstrated how production techniques can alter the volatile profiles of food products [12].

### 3.4. Cytotoxicity and Antiproliferative Assessment

**Figure 3:**
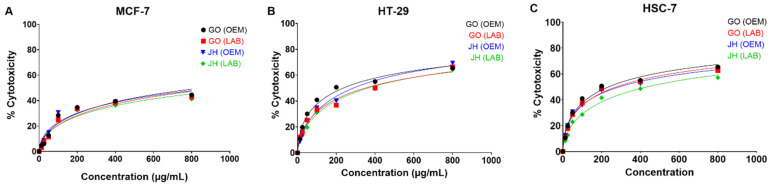
Cytotoxicity (%) of JH and GO in a dose-dependent manner. (**A**) MCF-7. (**B**) HT-29. (**C**) HSC-7. GO = Gang Om; JH = Jaew Hon; LAB = laboratory production; OEM = industrial production.

The results indicated that both JH OEM and GO OEM exhibited significantly greater cytotoxic effects against MCF-7 and HT-29 compared to their LAB-produced counterparts (JH LAB and GO LAB), as evidenced by higher maximum inhibition (Emax) percentages and lower IC_50_ values (Table 7). The effect is dose-dependent (Figure 3). The cytotoxic effects of GO OEM and JH OEM extracts were moderate for MCF-7, with Emax below 50% (44.51% and 43.17%) and IC_50_ values of 857.17 µg/mL and 938.00 µg/mL, respectively. However, the extracts showed significantly higher cytotoxicity toward HT-29, with GO OEM achieving an Emax of 71.88% and IC_50_ of 276.10 µg/mL, and JH OEM an Emax of 69.34% and IC_50_ of 286.77 µg/mL. For the HSC-7 cell line, GO OEM and GO LAB are more effective than JH OEM and JH LAB (Table 7).

The higher cytotoxicity may reflect bioactive contents (TPC and TFC) and volatile compound profiles. High levels of TPC and TFC have been extensively linked to anticancer properties [34,35].

**Figure 4:**
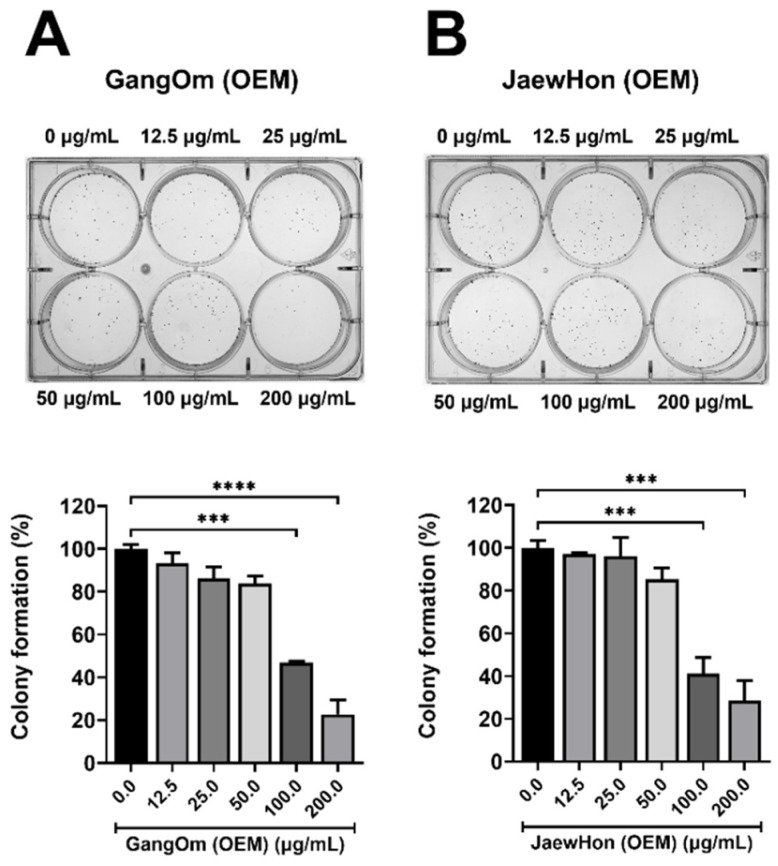
Colony formation (%) of HT-29 cells. (**A**) GO OEM. (**B**) JH OEM. GO = Gang Om; JH = Jaew Hon; OEM = Industrial production. The statistical significance levels included *p* < 0.001 (***), and *p* < 0.0001 (****).

The antiproliferative activity was further evaluated using a colony formation assay on HT-29 cells, where GO OEM demonstrated a significant dose-dependent inhibition of colony formation (Figure 4). At 50 µg/mL, a marked decline in both the number and size of colonies was detected, which became even more pronounced at 100 µg/mL, indicating strong antiproliferative activity. Conversely, JH OEM showed a less significant reduction in colony formation at the same concentrations, suggesting a comparatively weaker antiproliferative effect.

This disparity in antiproliferative potential between the two extracts may be due to different ingredients, bioactive contents (TPC and TFC), and volatile compound profiles. The improved anticancer potential of GO OEM may be attributable to the higher abundance of particular compounds, notably butanoic acid and benzoic acid, which are known for anticancer characteristics [36,37]. Recently, benzoic acid (BA) and its synthetic derivatives have emerged as promising anticancer agents due to their capacity to modulate key molecular pathways involved in cancer progression [37]. Sorbic acid, another preservative, is also prevalent in OEM, but it has not been reported to show an anticancer effect to date. Although beta-caryophyllene and myristicin are more prominent in JH OEM, the balanced profile of GO OEM, with increased butanoic acid, and benzoic acid, may support its stronger bioactivity. The cytotoxic effects on HT-29 cells may partially result from the synergistic interactions of these chemicals, as volatile organic compounds have the ability to influence cellular pathways associated with apoptosis and proliferation [38]. These findings underscore the importance of the volatile compound profile in determining bioactive potential and suggest that GO OEM’s composition favors anticancer activity against colon cancer cells.

### 3.5. Gene and Protein Expression Analysis

**Figure 5:**
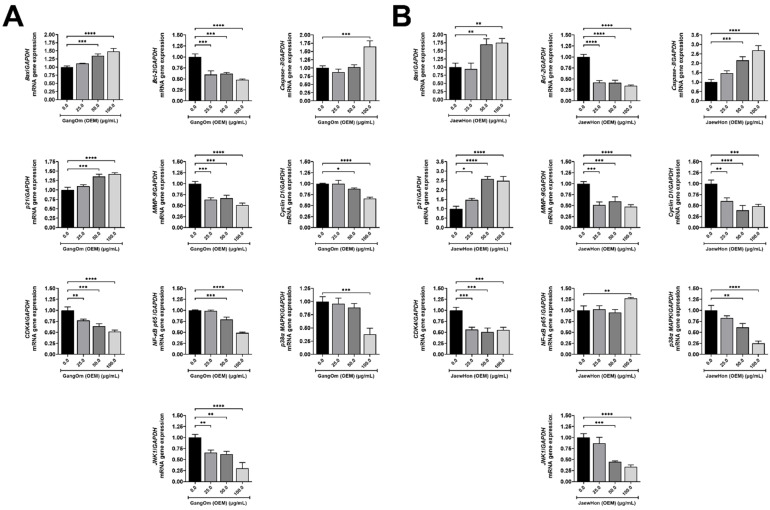
Gene expressions of HT-29 cells upon paste extract treatments. (**A**) GO OEM. (**B**) JH OEM. GO = Gang Om; JH = Jaew Hon; OEM = industrial production. The statistical significance levels included *p* < 0.05 (*), *p* < 0.01 (**), *p* < 0.001 (***), and *p* < 0.0001 (****).

The treatment with GO OEM led to a substantial overexpression of pro-apoptotic genes, specifically *Bax* and *Caspase-3* [39], in a dose-dependent way (Figure 5A). Moreover, the inflammatory marker *NF-κB p65* [40] was significantly downregulated.

Additionally, genes related to cell proliferation (*Cyclin D1*) and migration (*MMP-9*) [41] were also significantly downregulated at higher concentrations of GO OEM (Figure 5A), suggesting that GO OEM may inhibit these critical pathways involved in cancer progression. Furthermore, the expression levels of pathway-specific genes such as *p38 MAPK* and *JNK* were reduced, indicating a potential inhibition of stress-related signaling pathways that are frequently upregulated in colorectal cancer [42]. In contrast, JH OEM treatment demonstrated a weaker pro-apoptotic effect and a less significant suppression of the NF-κB pathway compared to GO OEM (Figure 5B). The expression of *Cyclin D1* and *MMP-9* showed only slight reductions, suggesting milder anticancer properties or alternative mechanisms of action. Additionally, the decreases in *p38 MAPK* and *JNK* expression were less pronounced, indicating a reduced impact on stress-induced and inflammatory signaling pathways.

**Figure 6:**
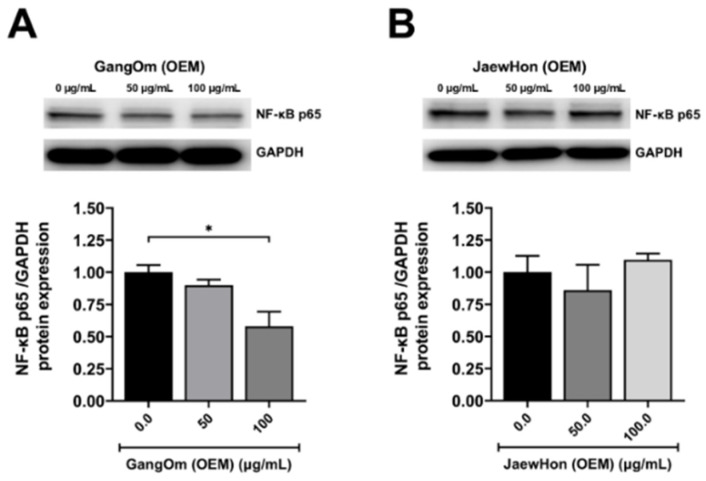
NF-κB protein expressions of HT-29 cells upon paste extract treatments. (**A**) GO OEM. (**B**) JH OEM. GO = Gang Om; JH = Jaew Hon; OEM = industrial production. The statistical significance level included *p* < 0.05 (*).

In HT-29, NF-κB p65 protein expression by Western blot analysis showed that GO OEM caused a dose-dependent decrease in NF-κB p65 expression (Figure 6). A significant reduction was observed at 50 µg/mL, with further decreases at 100 µg/mL, suggesting GO OEM may inhibit the NF-κB signaling pathway. In contrast, JH OEM showed no significant changes in NF-κB p65 levels across tested concentrations, indicating its effects might involve alternative mechanisms or other signaling pathways. NF-κB inhibition by GO OEM is noteworthy, as it is often overexpressed in colorectal cancer and linked to tumor survival [40].

The superior ability of GO OEM to suppress NF-κB protein expression in HT-29 cells compared to JH OEM may be due to its balance of bioactive compounds, which is able to modulate NF-κB signaling pathways by suppressing inflammation, promoting apoptosis, and disrupting cancer progression. These bioactive compositional differences may explain the enhanced anticancer efficacy of GO OEM and underscore its potential as a more effective therapeutic functional food for colon cancer cells.

## 4. Conclusions

This is the first study that demonstrates that food formulation and processing significantly influence the volatile compound profile and antioxidant and cytotoxic activities of JH and GO. The industrial production process enhanced the bioactive properties of the pastes, suggesting their potential as functional foods with health benefits. The integration of traditional culinary practices with modern food science not only preserves cultural heritage but also addresses contemporary health needs.

## Figures and Tables

**Table 1:** Ingredients (percent by weight) for laboratory-(LAB) and industrial (OEM)-scaled Jaew Hon (JH) and Gang Om (GO).

No.	Ingredients	JH OEM	JH LAB	GO OEM	GO LAB
1	Water	0	39	35.9	43
2	Fermented fish sauce	3.92	2	9.5	8
3	Shallots	4.7	4	9.24	8
4	Lemongrass	5.15	5	8.16	7
5	Toasted rice powder	1.93	2	7.36	6
6	Sweet basil leaves	0	0	5.23	5
7	Garlic	3.95	4	2.15	2
8	Chili powder	2.13	2	0	0
9	Chilli	0	0	3.45	2.5
10	Galangal	1.65	2	0	0
11	Wild betel leaves	4.5	5	2.15	5
12	Salt	1.96	4	1.89	1.5
13	Kaffir lime leaves	0.8	1	0.6	1
14	Chicken seasoning powder	4.5	0	0.8	1
15	Dill	0	0	7.76	5
16	Hoary basil	4.5	5	5.23	5
17	Braised soup	42.93	10	0	0
18	Coconut sugar	1.9	2	0	0
19	Tamarind juice	1.9	2	0	0
20	Black pepper	4.5	1	0	0
21	Sawtooth coriander	4.5	5	0	0
22	Vietnamese coriander	4.5	5	0	0
23	Fish sauce	0	0	0.5	0
24	Potassium sorbate INS 202	0.04	0	0.04	0
25	Sodium benzoate INS 211	0.04	0	0.04	0
	Total	100	100	100	100

**Table 2:** Gene-specific primers for real-time PCR.

Gene	Gene Role	Primer	Sequence (5′-3′)	Size (bp)
*GAPDH*	Housekeeping gene	Forward	GGATTTGGTCGTATTGGGCG	115
		Reverse	TCCCGTTCTCAGCCATGTAG	
Apoptotic pathway				
*Bax*	Promote apoptosis	Forward	GAGCAGCCCAGAGGCG	276
		Reverse	AGCTGCCACTCGGAAAAAGA	
*Bcl-2*	Inhibit apoptosis	Forward	ATGTGTGTGGAGAGCGTCAA	135
		Reverse	ATCACCAAGTGCACCTACCC	
*Caspase-3*	Execute apoptosis via cleavage	Forward	GTGCTATTGTGAGGCGGTTG	271
		Reverse	GTTTCCCTGAGGTTTGCTGC	
*p38α MAPK*	Regulate apoptosis via stress signaling	Forward	AACAGGATGCCAAGCCATGA	229
		Reverse	CATAAGGATCGGCCACTGGT	
*JNK1*	Activate apoptosis via stress response	Forward	CTCTCCTTTAGGTGCAGCAGT	102
		Reverse	GAGGCCAAAGTCGGATCTGT	
Cell cycle regulation pathway				
*p21*	Cell cycle arrest	Forward	CCCAACGCACCGAATAGTTAC	167
		Reverse	GAAAACTCCCCAGGAAGCCT	
*Cyclin D1*	Drive cell cycle	Forward	GCTGTAGTGGGGTTCTAGGC	297
		Reverse	AGCGTATCGTAGGAGTGGGA	
*CDK4*	Regulate cell cycle	Forward	GTATGGGGCCGTAGGAACC	113
		Reverse	AGGCAGAGATTCGCTTGTGT	
Inflammation and survival pathway				
*NF-κB p65*	Mediate inflammation, apoptosis resistance	Forward	CTGCACTGTGGGGTCACAT	114
		Reverse	GGACACTTGAATCAGCAGGC	
Matrix remodeling and tumor progression pathway				
*MMP-9*	Matrix, remodeling, Apoptosis regulation	Forward	TATGACATCCTGCAGTGCCC	111
		Reverse	TTGTATCCGGCAAACTGGCT	

**Table 3:** Physicochemical attributes of the four pastes in laboratory and industrial production.

Sample	pH	TDS (°Brix)	a_w_	L*	a*	b*
GO OEM	5.28 ± 0.04 ^a^	7.83 ± 0.15 ^c^	0.94 ± 0.00 ^a^	31.43 ± 0.56 ^a^	1.03 ± 0.13 ^c^	7.90 ± 0.20 ^a^
GO LAB	5.21 ± 0.01 ^b^	6.67 ± 0.31 ^d^	0.92 ± 0.00 ^b^	30.67 ± 0.27 ^b^	1.03 ± 0.08 ^c^	8.33 ± 0.27 ^a^
JH OEM	5.13 ± 0.01 ^c^	11.57 ± 0.21 ^a^	0.91 ± 0.00 ^c^	29.57 ± 0.21 ^c^	1.82 ± 0.13 ^a^	8.39 ± 0.66 ^a^
JH LAB	4.92 ± 0.02 ^d^	10.73 ± 0.25 ^b^	0.90 ± 0.00 ^d^	29.68 ± 0.14 ^c^	1.46 ± 0.06 ^b^	8.66 ± 0.42 ^a^

Different superscripts within the same column denote statistically significant differences (*p* < 0.05). TDS = total dissolved solid; a_w_ = water activity; GO = Gang Om; JH = Jaew Hon; LAB = laboratory production; OEM = industrial production.

**Table 4:** Antioxidant activity and bioactive contents of key herbal ingredients used in JH and GO.

Ingredient	DPPH (mg AAE/100 g DW)	FRAP (mg FeSO_4_/100 g DW)	TPC (mg GAE/100 g DW)	TFC (mg QE/100 g DW)
Dill	95.95 ± 2.88 ^f^	592.19 ± 4.65 ^b^	287.03 ± 0.13 ^c^	213.9 ± 6.96 ^e^
Vietnamese coriander	33.18 ± 0.08 ^g^	424.46 ± 6.29 ^e^	171.14 ± 0.18 ^f^	118.01 ± 1.26 ^g^
Hoary basil	148.9 ± 6.58 ^d^	606.84 ± 6.93 ^b^	252.51 ± 1.64 ^d^	333.68 ± 1.51 ^b^
Sawtooth coriander	18.65 ± 1.36 ^h^	420.77 ± 1.09 ^e^	50.29 ± 0.17 ^i^	82.74 ± 1.31 ^g^
Wild betel leaves	125.25 ± 4.97 ^e^	556.56 ± 11.82 ^c^	230.26 ± 0.21 ^e^	364.87 ± 4.38 ^a^
Chili	1419.51 ± 54.38 ^a^	855.43 ± 28.01 ^a^	1313.92 ± 17.28 ^a^	237.73.41 ± 1.08 ^d^
Kaffir lime leaves	558.5 ± 23.33 ^b^	520.46 ± 45.94 ^c^	123.61 ± 0.14 ^h^	205.84 ± 9.89 ^e^
Garlic	129.54 ± 1.68 ^e^	152.12 ± 10.37 ^g^	129.55 ± 0.47 ^g^	118.58 ± 0.69 ^f^
Shallot	264.13 ± 0.01 ^c^	486.06 ± 5.61 ^d^	381.87 ± 18.16 ^b^	86.53 ± 2.32 ^g^
Sweet basil leaves	101.76 ± 6.28 ^f^	385.42 ± 1.15 ^f^	286.53 ± 0.26 ^c^	274.37 ± 3.99 ^c^

Different superscripts within the same column denote statistically significant differences (*p* < 0.05).

**Table 5:** Antioxidant action and bioactive components of JH and GO.

Sample	DPPH (mg AAE/100 g)	FRAP (mg FeSO_4_/100 g)	TPC (mg GAE/100 g)	TFC (mg QE/100 g)
GO OEM	82.76 ± 3.54 ^b^	247.43 ± 4.24 ^c^	430.49 ± 14.21 ^a^	345.57 ± 5.30 ^a^
GO LAB	49.51 ± 0.10 ^c^	115.22 ± 0.4 ^d^	259.81 ± 1.94 ^c^	124.69 ± 5.21 ^c^
JH OEM	84.41 ± 0.70 ^b^	809.55 ± 6.79 ^a^	352.85 ± 8.17 ^b^	191.44 ± 1.40 ^b^
JH LAB	96.25 ± 1.32 ^a^	742.5 ± 4.91 ^b^	433.50 ± 0.10 ^a^	89.67 ± 6.64 ^d^

Different superscripts within the same column denote statistically significant differences (*p* < 0.05). GO = Gang Om; JH = Jaew Hon; LAB = laboratory production; OEM = industrial production.

**Table 6:** Volatile compounds (% relative abundance) in four pastes.

Compound	CAS Number	% Relative Abundance			
		JH OEM	JH LAB	GO OEM	GO LAB
1. Acetic acid	64-19-7	0.42	0.45	0.71	0.83
2. Butanoic acid	107-92-6	0.27	0.39	1.33	1.18
3. 1R-alpha-Pinene	7785-70-8	0.37	nd	nd	nd
4. (-)-beta-Pinene	18172-67-3	0.98	0.12	0.09	0.07
5. beta-Myrcene	123-35-3	0.25	0.15	0.10	0.14
6. alpha-Phellandrene	99-83-2	0.48	0.19	0.83	0.55
7. 3-Carene	13466-78-9	2.64	0.32	0.25	0.19
8. omicron-Cymene	527-84-4	0.19	0.06	0.20	0.10
9. D-Limonene	5989-27-5	2.45	0.69	0.66	0.50
10. Benzeneacetaldehyde	122-78-1	0.04	0.12	0.09	0.11
11. beta-Ocimene	13877-91-3	0.06	0.14	0.10	0.14
12. Diallyl disulphide	2179-57-9	0.05	0.13	0.03	0.09
13. beta-Linalool	126-90-9	0.45	0.46	0.27	0.25
14. Sorbic Acid	110-44-1	7.52	0.00	18.81	0.00
15. Isopulegol	89-79-2	0.19	0.61	0.25	0.37
16. Citronellal	106-23-0	0.27	1.34	0.34	0.93
17. Terpinen-4-ol	562-74-3	0.25	0.15	0.12	0.06
18. alpha-Terpineol	98-55-5	0.48	0.12	0.15	0.15
19. Estragole	140-67-0	4.23	6.67	6.15	5.89
20. Benzoic acid	65-85-0	3.83	nd	5.90	nd
21. Citronellol	106-22-9	0.56	1.15	0.62	0.92
22. beta-Citral	5392-40-5	0.11	0.95	0.10	0.38
23. trans-Geraniol	106-24-1	0.27	1.08	0.66	1.47
24. Phenol, 4-(2-propenyl)-	97-53-0	0.13	nd	nd	nd
25. alpha-Citral	141-27-5	0.16	1.62	0.18	0.68
26. 2-Hydroxycineole	470-67-7	0.35	nd	nd	nd
27. Piperonal	120-57-0	0.47	nd	nd	nd
28. exo-2-Hydroxycineole acetate	16409-46-4	0.57	2.28	0.23	0.14
29. alpha-Cubebene	17699-14-8	0.31	0.20	0.21	0.19
30. Citronellol acetate	150-84-5	0.66	1.96	0.67	1.12
31. 8-Hydroxymenthol	1197-01-9	0.89	5.77	2.19	2.79
32. Copaene	3856-25-5	1.79	0.80	1.41	0.90
33. Geranyl acetate	105-87-3	0.49	2.29	0.65	1.09
34. (-)-beta-Elemene	515-13-9	0.80	1.68	1.08	1.60
35. Methyleugenol	93-15-2	0.19	0.69	0.26	0.27
36. Dodecanal	112-54-9	0.94	0.47	nd	nd
37. beta-Caryophyllene	87-44-5	17.31	8.58	5.89	6.31
38. alpha-Bergamotene	17699-05-7	1.06	3.38	2.38	3.48
39. alpha-Caryophyllene	6753-98-6	1.53	1.60	1.13	1.42
40. cis-beta-Farnesene	18794-84-8	0.52	2.82	0.68	1.17
41. beta-Cubebene	13744-14-8	0.61	1.27	0.85	1.59
42. beta-Eudesmene	20307-84-0	0.69	1.20	0.45	0.67
43. beta-Maaliene	473-04-1	0.19	0.69	0.30	0.96
44. delta-Guaiene	3691-12-1	0.22	0.76	0.36	0.60
45. beta-Bisabolene	495-61-4	0.47	1.09	0.35	0.44
46. gamma-Cadinene	39029-41-9	0.41	1.85	0.88	2.36
47. Myristicin	607-91-0	24.50	3.99	18.09	4.27
48. 1,4,7,-Cycloundecatriene, 1,5,9,9-tetramethyl-, Z,Z,Z-	489-41-8	0.26	0.28	1.11	1.67
49. Elemol	639-99-6	0.32	0.26	0.26	0.64
50. Elemicin	487-11-6	1.31	nd	0.71	nd
51. Nerolidol	7212-44-4	0.76	1.72	0.80	1.24
52. Caryophyllene oxide	1139-30-6	1.46	0.44	0.35	0.43
53. alpha-Eudesmol	473-15-4	0.24	0.58	0.35	0.92
54. Selina-6-en-4-ol	23123-36-6	1.35	7.99	4.28	17.02
55. Spathulenol	6750-60-3	3.76	nd	2.09	nd
56. epi-alpha-Cadinol	481-34-5	0.77	2.20	1.75	3.00
57. alpha-Cadinol	481-34-5	0.50	nd	nd	3.82
58. alpha-Bisabolol	515-69-5	0.15	2.61	nd	nd
59. Juniper camphor	464-45-9	0.09	0.97	0.36	1.95
60. Farnesol	4602-84-0	0.10	0.33	0.16	0.94
61. 1-Decanol, 2-hexyl-	2425-77-6	0.63	1.72	2.34	1.90
62. Phytol, acetate	7541-49-3	0.20	0.81	1.39	1.36
63. 2-Pentadecanone, 6,10,14-trimethyl-	1937-63-1	0.06	0.18	0.11	0.10
64. Hexadecanoic acid, methyl ester	112-39-0	1.27	0.24	0.13	0.05
65. n-Hexadecanoic acid	57-10-3	0.11	nd	nd	0.10
66. n-Butyl laurate	123-95-5	0.09	0.49	0.58	0.76
67. Hexadecanoic acid, ethyl ester	628-97-7	0.11	0.00	0.09	0.07
68. Isopropyl palmitate	142-91-6	0.04	0.09	0.05	0.05
69. Linoleic acid, methyl ester	112-63-0	0.74	0.03	0.05	nd
70. Oleic acid, methyl ester	112-62-9	0.53	nd	nd	nd
71. Phytol	150-86-7	0.21	0.06	0.45	0.31
72. Methyl stearate	112-61-8	0.06	nd	nd	nd
73. Ethyl Oleate	111-62-6	0.04	nd	nd	nd
74. Hexadecanoic acid, butyl ester	5923-95-1	0.08	0.04	0.07	0.06

GO = Gang Om; JH = Jaew Hon; LAB = laboratory production; OEM = industrial production; nd = not detected.

**Table 7:** Cytotoxicity (%) and IC_50_ of JH and GO.

Sample	MCF-7	HT-29	HSC-7	MCF-7	HT-29	HSC-7
	Emax (%)	Emax (%)	Emax (%)	IC_50_ (µg/mL)	IC_50_ (µg/mL)	IC_50_ (µg/mL)
GO OEM	44.51 ± 0.19 ^aC^	71.88 ± 0.17 ^aA^	65.41 ± 0.18 ^aB^	857.17 ± 3.33 ^aC^	276.10 ± 1.08 ^aB^	235.13 ± 1.71 ^aA^
GO LAB	42.91 ± 0.00 ^cC^	66.32 ± 0.20 ^cA^	62.77 ± 0.18 ^bB^	960.30 ± 3.10 ^cC^	348.33 ± 0.99 ^cA^	274.90 ± 1.54 ^bB^
JH OEM	43.17 ± 0.11 ^bC^	69.34 ± 0.44 ^bA^	62.47 ± 0.10 ^bB^	938.00 ± 1.56 ^bB^	286.77 ± 2.17 ^bA^	290.53 ± 2.45 ^cA^
JH LAB	41.21 ± 0.19 ^dC^	64.40 ± 0.20 ^dA^	57.22 ± 0.36 ^cB^	1123.00 ± 2.00 ^dC^	361.73 ± 2.20 ^dA^	433.60 ± 2.17 ^dB^

Different superscripts ^a,b,c,d^ in the same columns and ^A,B,C^ in the rows indicate statistical significance (*p* < 0.05). GO = Gang Om; JH = Jaew Hon; LAB = laboratory production; OEM = industrial production.

## Data Availability

The original contributions presented in this study are included in the article. Further inquiries can be directed to the corresponding author.
